# Basic Emotions in the Nencki Affective Word List (NAWL BE): New Method of Classifying Emotional Stimuli

**DOI:** 10.1371/journal.pone.0132305

**Published:** 2015-07-06

**Authors:** Małgorzata Wierzba, Monika Riegel, Marek Wypych, Katarzyna Jednoróg, Paweł Turnau, Anna Grabowska, Artur Marchewka

**Affiliations:** 1 Laboratory of Brain Imaging, Neurobiology Centre, Nencki Institute of Experimental Biology, Warsaw, Poland; 2 Laboratory of Psychophysiology, Department of Neurophysiology, Nencki Institute of Experimental Biology, Warsaw, Poland; 3 University of Social Sciences and Humanities, Warsaw, Poland; University of Akron, UNITED STATES

## Abstract

The Nencki Affective Word List (NAWL) has recently been introduced as a standardized database of Polish words suitable for studying various aspects of language and emotions. Though the NAWL was originally based on the most commonly used dimensional approach, it is not the only way of studying emotions. Another framework is based on discrete emotional categories. Since the two perspectives are recognized as complementary, the aim of the present study was to supplement the NAWL database by the addition of categories corresponding to basic emotions. Thus, 2902 Polish words from the NAWL were presented to 265 subjects, who were instructed to rate them according to the intensity of each of the five basic emotions: happiness, anger, sadness, fear and disgust. The general characteristics of the present word database, as well as the relationships between the studied variables are shown to be consistent with typical patterns found in previous studies using similar databases for different languages. Here we present the Basic Emotions in the Nencki Affective Word List (NAWL BE) as a database of verbal material suitable for highly controlled experimental research. To make the NAWL more convenient to use, we introduce a comprehensive method of classifying stimuli to basic emotion categories. We discuss the advantages of our method in comparison to other methods of classification. Additionally, we provide an interactive online tool (http://exp.lobi.nencki.gov.pl/nawl-analysis) to help researchers browse and interactively generate classes of stimuli to meet their specific requirements.

## Introduction

The protracted and acrimonious debate on the nature of emotion has left contemporary researchers with no clear guidelines to follow. Two distinct camps have emerged, with quite different views on what conceptual model of emotions works best [1, 2].

Some authors prefer to explain emotional mechanisms in terms of *affective dimensions* [3], what could be seen as building blocks underlying emotional experience [4]. In this approach, each variable is a continuous interval between two extremes representing opposite states [5]. Since the variables are assumed to be independent of each other, any value in one dimension can be combined with any value in another dimension. Consequently, affective dimensions are assumed to furnish us with a complete description of every emotional event, the totality of such possible events being modelled by a *multidimensional affective space* [3]. In other words, one can identify a particular emotional state by specifying a vector (set of coordinates) in a multidimensional space [1, 3, 5, 6]. The dimensions most often identified as crucial to the explanation of emotion are: *valence* (also: *pleasure*, *evaluation*, *positivity*) and *arousal* (also: *activity*, *activation*, *excitation*, *intensity*, *emotional charge*). It was found in many studies that these two dimensions were sufficient to characterize differences between emotional events, and attempts to add more dimensions to the affective space have been less successful and thus gained little recognition [6–9].

Advocates of the competing framework argue that each emotional event can be decomposed into several elementary states, and differences between such events depend on different proportions of these elementary states. To characterize emotional diversity, these researchers invoke discrete categories, such as *basic emotions* (also: *primary*, *fundamental*, *modal emotions*; see: [2, 10]). The concept of basic emotions itself has been interpreted in various ways and thus different theories posit different numbers of such basic states [2, 11, 12]. Criteria employed to identify such basic states include: unique neural basis, unique physiological response pattern or unique behavioural expression [4, 10]. To categorize emotions, semantic concepts drawn from natural language are used in this approach. Since categories provided by language appear to correspond to specific behavioural or physiological response patterns, such language-based differentiation of emotional states seems reasonable [10].

The controversy over the nature of emotion has been recently re-evaluated, leading to attempts to combine both perspectives in a common theoretical framework. It has been pointed out that the two approaches, dimensional and discrete, are not contradictory and should rather be considered complementary [1, 13]. Strong evidence for the usefulness of both perspectives in the study of emotions has been provided by behavioural science, as well as by neuroscience. While a large body of research supports the dimensional theory of emotion [13, 14], the discrete approach should not be neglected either [15]. There have been many attempts to identify brain regions activated specifically in response to a given emotion. Nevertheless, it is not clear what conclusions can be drawn from these studies [1]. Although technical progress in neuroscience will undoubtedly produce more fine-grained data, it may also be crucial to revise the way research questions are formulated. Recent publications emphasized the importance of the combined approach [7, 9, 16] and suggested that the simultaneous control for both dimensional and discrete variables is a proper direction for further research. Studies conducted within such a combined approach have successfully documented that discrete emotion variables improved our understanding of affective processing [17–19]. In particular, it has been suggested that the two approaches reveal processes that differ in their temporal characteristics, indicating sequential processing of affective information [17]. Moreover, recent neuroimaging research provided evidence, that the dimensional and discrete features could be regarded as belonging to different domains of affective processing and thus involve separate neural networks [18].

In order to establish standards in emotion research, certain solutions have been proposed. These involve the use of *affective databases* (*datasets* / *lists* / *systems*). Such databases serve as sources of standardized stimuli that can be controlled for several potentially significant affective characteristics. Most affective databases offer visual stimuli, both non-verbal (pictures) and verbal (words). The former include, for instance, the International Affective Picture System (IAPS; [20]) and the Nencki Affective Picture System (NAPS; [21]). Examples of the latter are the Nencki Affective Word List (NAWL; [22]), the Berlin Affective Word List—Reloaded (BAWL-R; [23]) and the Affective Norms for English Words (ANEW; [24]).

Unlike non-verbal material, universal and useful in studies of populations from different cultures, verbal stimuli are by nature culturally biased. Thus, applicability of the latter is limited by linguistic differences between populations. Affective word databases are already available in several languages: English [9, 24–26], German [7, 23], Spanish [8], Finish [25], Portuguese [27], Dutch [28], French [29] and Italian [30]. Similarly, databases for the Polish language have been recently introduced: the Affective Norms for 1,586 Polish Words (ANPW; [31]) and the Nencki Affective Word List (NAWL, [22]). However, most databases provide only general affective characteristics of words, such as valence (negative–positive) and arousal (low–high). Only two have been supplemented by the addition of information on word features corresponding to basic emotions [7, 9] or other variables presumed to influence affective processing of words (e.g. embodied cognition features, aestetic features; [19]). Importantly, recent research emphasizes that verbal material processing should be studied not only in terms of *cold* information processing, but would also benefit from a detailed investigation of *hot*, i.e. affective and aestetic processing [19]. Empirical evidence gathered to date provides a rationale for collecting such norms in other languages, providing important motivation for the present work.

In order to furnish researchers with emotional stimuli, suitable for studying emotion from both perspectives simultaneously, we supplemented the NAWL with happiness, anger, sadness, fear and disgust ratings. Moreover, we investigate the extent to which the ratings obtained from the present study correspond to the ratings from the original German DENN-BAWL study [7]. Importantly, we decided to refrain from making any direct comparisons between the present study and the ANEW study [9] since the BAWL and ANEW overlap only in a small part [32]. Additionally, we evaluate existing classifications of words as to their usefulness for identifying words that elicit basic emotions. We also propose a new classification with this property.

## Method

### Materials

In the present study we used 2902 Polish words (1676 nouns, 614 verbs, 612 adjectives) from the NAWL [22], a cultural adaptation of the German BAWL-R [23] database. The experimental material included in the NAWL database contains both emotional and neutral words, and provides information on their valence, arousal and imageability, as well as several linguistic features: number of letters, frequency of use in Polish, as well as grammatical category. Lexical properties have been obtained from the balanced National Corpus of Polish Language (NKJP; [33]), as well as from SUBTLEX-PL dataset based on movie subtitles [34].

### Participants

265 healthy subjects (132 female, 133 male) aged 18–60 (M = 23.4; SD = 5.9) were recruited for the study. All participants held Polish citizenship and indicated Polish as their first language. Most of the participants were college students or young graduates from a wide range of faculties and departments of several universities and schools in Warsaw. Several recruitment channels were employed, including mass mailings arranged by student unions, social media and personal communication. Subjects received financial gratification for their participation in the amount of PLN 30 (approximately EUR 7). A local research ethics committee at the University of Social Sciences and Humanities (Komisja ds. Etyki Badań Naukowych Wydziału Psychologii SWPS) approved the experimental protocol of the study.

It should be noted that the sample in the present study was matched for its demographic description with the sample in [22]. Thus, the data sets collected in the NAWL and NAWL BE studies contain ratings obtained from similar groups of participants.

### Procedure

Prior to the assessment task, participants were asked to sign the informed consent form. They were also assured that they could quit the experiment at any point of time without revealing their reasons. They were instructed not to disclose any information about the study for at least 30 days. Subjects worked in groups (of up to 8 persons), with each person rating words individually on a separate computer station, using a web browser. An assessment platform running on a local server was used to collect the ratings. Participants had unlimited time to complete the task, with a single experimental session lasting approximately 60 minutes. An optional short break was allowed.

The design of the rating procedure was based on procedures previously used for German and English databases constructed within the discrete emotion framework (Discrete Emotion Norms for Nouns–Berlin Affective Word List, DENN-BAWL, [7]; Affective Norms for English Words, ANEW, [9]). The full list of 2902 words was split into 10 sets, identical to those used in the previous study [22]. Each participant rated one set of 291 words. Each word was rated by approximately 26–27 subjects.

Before attempting the assessment task, subjects read instructions explaining the general purpose of the study and the rating scales. Participants were encouraged to indicate their immediate, spontaneous reaction to words. The full text of the instruction in Polish, as well as its English translation, can be found in S1 Appendix. Subjects were able to return to the instruction at any time during the session and resume work. An assistant was present during the whole experiment to answer any questions concerning the task.

During the rating task, words were displayed and rated one at a time. Initially a single word in full-screen mode was presented for 1 second, after which the rating scales appeared along with the same word, set in a smaller font in the upper part of the screen. Participants were asked to assess the intensity of basic emotions elicited by words on 7-point scales corresponding to happiness, anger, sadness, fear and disgust. Subjects used a standard computer mouse to enter their ratings. As soon as a word was rated on every scale, the subject was able to advance to the next screen, on which the subsequent word was displayed.

## Results

### Data preprocessing

Given that each of the 265 participants rated 291 words on 5 basic emotion scales, the total number of ratings amounted to 385 575. Ratings from two participants (1 female, 1 male) were discarded due to misinterpretation of instructions.

### Reliability analysis

To verify the internal consistency of the NAWL ratings a split-half reliability estimation was performed [29, 30]. For this purpose, subjects were subdivided into odd/even entries according to the order in which they participated in the experiment. Within each subsample, the mean for each basic emotion for each word was calculated. Pairwise Pearson’s correlation coefficients between mean ratings obtained from the respective subsamples were then calculated and adjusted using the Spearman-Brown formula. A corresponding procedure was then performed for the group of females and males separately. All correlations were significant at the .01 level (two-tailed). The obtained reliability coefficients appeared to be sufficiently high (Table 1), suggesting that the ratings are consistent both for the whole sample and for groups of females and males.

**Table 1:** Split-half reliabilities for each variable calculated for the whole sample, as well as for the group of females and males separately.

	Whole sample	Females	Males
**Happiness**	0,95	0,89	0,89
**Anger**	0,91	0,86	0,83
**Sadness**	0,92	0,88	0,84
**Fear**	0,92	0,85	0,81
**Disgust**	0,91	0,83	0,82

### General description of the database

For each word and for each discrete category—happiness, anger, sadness, fear and disgust—the mean (*M*), standard deviation (*SD*), minimum and maximum were calculated. The calculations were done for the whole sample, as well as for females and males separately. The general overview of the variables included in the NAWL can be found in Table 2. Descriptive statistics are given for the discrete emotion categories (happiness, anger, sadness, fear and disgust) used in the present study, as well as for the ratings of valence, arousal and imageability dimensions obtained from previous study [22].

**Table 2:** Descriptive statistics (*M*–mean, *SD*–standard deviation, *min*–minimum, *max*–maximum) for all variables in the NAWL calculated for the whole sample for each of n = 2902 Polish words.

	*M*	*SD*	*min*	*max*	range of the scale
**Valence**	0.17	1.18	-2.73	2.76	-3–3
**Arousal**	2.38	0.54	1.11	4.27	1–5
**Imageability**	5.60	0.70	2.67	6.89	1–7
**Happiness**	2.94	1.35	1	6.74	1–7
**Anger**	2.21	0.98	1	5.88	1–7
**Sadness**	2.16	0.99	1	6.35	1–7
**Fear**	2.45	1.03	1	6.15	1–7
**Disgust**	1.96	0.90	1	5.92	1–7

**Fig 1:**
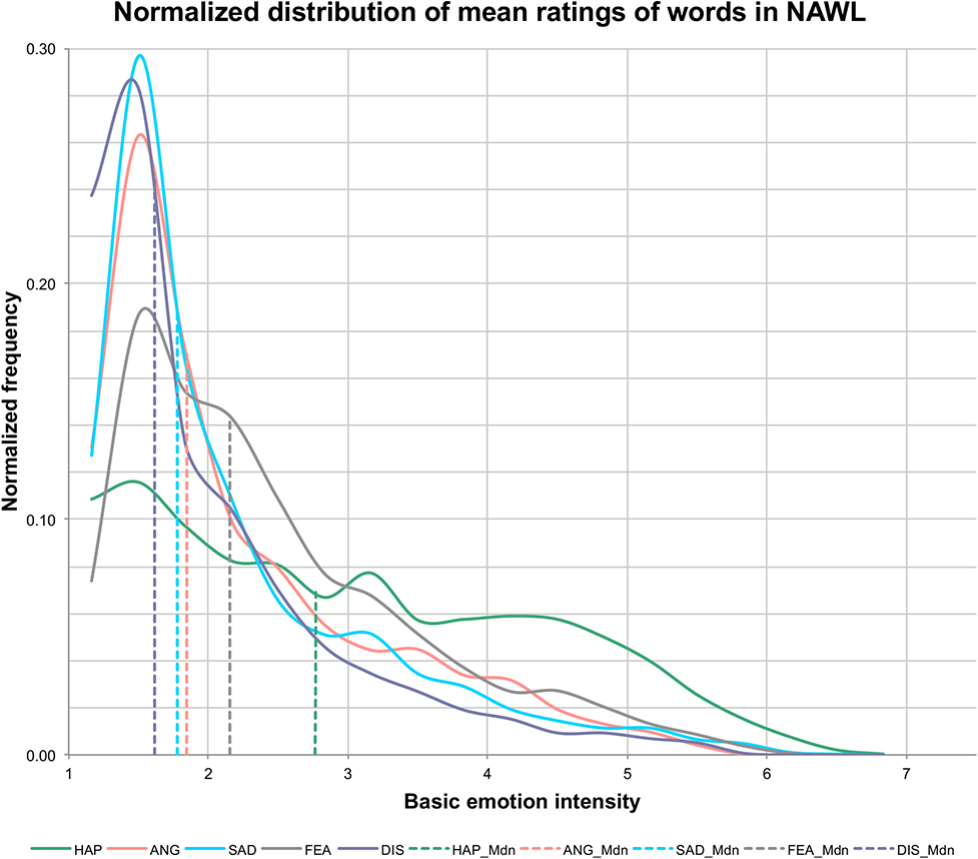
Normalized frequency distribution of mean ratings for the basic emotions included in the NAWL (n = 2902). Dotted lines represent median values of the respective distributions.

To analyse the distribution of mean ratings for each basic emotion over the set of 2902 words, we divided the full range (1–7) into 18 equal bins. For each bin, the number of means falling within the bin range was calculated for each basic emotion separately. Frequencies obtained in this way (normalized by dividing them by the number of words in the database) were plotted (Fig 1) following [35]. Dotted lines indicate median values (*Mdn*) in each emotion category. The plot makes it immediately clear that lower ratings were much more frequent, irrespective of emotion category. In particular, the frequencies of words rated below 2 were: 32% for happiness, 57% for anger, 59% for sadness, 42% for fear and 65% for disgust. The plot also shows that happiness ratings are much more dispersed, covering more uniformly the whole range, indicating that it is relatively easier to find words eliciting higher values of happiness than of other basic emotions.

### Between-variables relationships

Pearson’s correlation coefficients were calculated to examine relationships between the studied variables. Correlation analysis yielded a characteristic pattern of relationships among the basic emotion categories. Happiness correlated negatively with all the other categories (*r* = -.66; *p* < 0.01 for anger; *r* = -.60; *p* < 0.01 for sadness; *r* = -.57; *p* < 0.01 for fear; *r* = -.66; *p* < 0.01 for disgust). The remaining emotion categories (anger, sadness, fear and disgust) were positively correlated to each other. Ratings from the present study were also correlated with ratings of valence and arousal obtained from previous study [22]. As far as correlation with valence is concerned, it was strong and positive for happiness (*r* = .91; *p* < 0.01), and also strong but negative for the remaining emotion categories (*r* = -.80; *p* < 0.01 for anger; *r* = -.74; *p* < 0.01 for sadness; *r* = -.70; *p* < 0.01 for fear; *r* = -.77; *p* < 0.01 for disgust). Correlations between discrete categories and arousal were much weaker. Of all discrete categories, happiness appeared to be least related to arousal (*r* = .13; *p* < 0.01). On the other hand, fear was found to be the emotion category most strongly correlated with arousal (*r* = .46; *p* < 0.01). The respective statistics can be found in Table 3.

**Table 3:** Correlations between variables included in the NAWL database (n = 2902). All correlations were significant at the .01 level (two-tailed).

	Happiness	Anger	Sadness	Fear	Disgust
**Happiness**	1				
**Anger**	-.66	1			
**Sadness**	-.60	.78	1		
**Fear**	-.57	.72	.78	1	
**Disgust**	-.66	.78	.66	.66	1
**Valence**	.91	-.80	-.74	-.70	-.77
**Arousal**	.13	.40	.39	.46	.30

### Classification of words associated with basic emotions

In the present study, subjects rated words on five scales corresponding to the five basic emotions (happiness, anger, sadness, fear and disgust). In other words, they were asked whether, and how strongly, a given word elicited each of these basic emotions. However, effective experimental manipulation requires stimuli that evoke one kind of emotional experience specifically, without eliciting the other emotional states. In the literature several criteria have been proposed for the classification of stimuli in affective databases [7,16]. Here we briefly review these classification methods and discuss their merits in relation to the new method proposed here.

The simplest classification method was introduced as *liberal* in [7]. In this method, a word is considered to belong to a particular emotion category if its mean rating for this category is higher than for the remaining categories. Although this criterion is simple and intuitive, its major drawback is that it is very sensitive to errors in the mean estimation. Thus, if the means for different categories happen to be close, even a negligible difference between means has decisive impact on classification. A word could therefore be misclassified as eliciting only a certain emotion, while in fact it elicits two or more different emotions at comparable levels. Applied to the NAWL data, this method classified 2861 out of 2902 words as related to happiness (1669), anger (330), sadness (215), fear (499) and disgust (148).

The second method introduced in [7] as *conservative* is a modification of the highest mean criterion described above. In this approach, classification is also based on differences between means for different basic emotions, but these differences are required to exceed one *SD*. Using this criterion, we assigned 816 out of 2902 words to the following categories: happiness (771), anger (7), sadness (13), fear (15) and disgust (10).

The last criterion, introduced in [16], is similar to the *conservative* criterion described above [7]. However, the minimum distance separating the mean ratings for the dominant emotion and for the remaining emotions is based on confidence intervals. More exactly, to determine how much the *M*s must differ from each other for a given word to be assigned to a single basic emotion, we construct confidence intervals (CI) around each *M*. Having calculated the CI for each basic emotion (for a given word), we can decide whether the word should be assigned to a *single* dominant basic emotion (if there is no overlap between the CI for the dominant emotion and for the remaining emotions) or whether it should be assigned to a *blended* category (if the CIs of two or three categories, including the dominant one, overlap) or whether the word is *undifferentiated* with respect to basic emotions. Having applied this classification criterion to the NAWL data, we were able to assign 1289 words to a single emotion: happiness (1150), anger (23), sadness (35), fear (57) and disgust (24). Additionally, we identified 158 blended emotion words, which elicited several emotions at a similar level. 1455 out of 2902 words remained undifferentiated.

A comparison of the classification criteria as applied to the NAWL database is shown in Table 4. The above criteria are all based on the comparison of means, differing only in how restrictive they are in assigning words to basic emotion classes. Clearly, this means that these classification schemes are mutually consistent. However, they tend to classify words either too broadly or too narrowly, making the trade-off between quality and number of experimental stimuli immediately apparent. Moreover, the construction of these criteria make it impossible for the researcher to adjust the classification to the requirements of a particular experimental design. Hence, they are not entirely satisfactory.

**Table 4:** Number of NAWL words (n = 2902) representing distinct basic emotions as classified with different methods [7, 16]. For Euclidean distance (ED) based classification method following threshold values were used: 2.5 for happiness, 5.5 for anger, sadness, fear, disgust; 2.5 for the neutral class.

	Briesemeister et al. (2011)—*liberal*	Briesemeister et al. (2011)—*conservative*	Mikels et al. (2005)	ED based classification method
**Happiness**	1669	771	1150	147
**Anger**	330	7	23	98
**Sadness**	215	13	35	64
**Fear**	499	15	57	163
**Disgust**	148	10	24	48
**Neutral**	-	-	-	219
***blended***	-	-	158	-
***Undifferentiated /unclassified***	41	2 086	1455	2163

To make the NAWL database more convenient to use, we provide yet another classification criterion. Given a vector of 5 individual ratings, obtained from a subject for a given word, we calculate its Euclidean distance to five “extreme” points, representing pure basic emotions: (7,1,1,1,1) for happiness, (1,7,1,1,1) for anger, (1,1,7,1,1) for sadness, (1,1,1,7,1) for fear and (1,1,1,1,7) for disgust. We use (1,1,1,1,1) to represent the neutral state. These distances are then averaged over the whole sample for a given word and a given emotion to produce a measure of how close the word is to each of the extreme points. Although the neutral class was neglected in previous classifications [7, 16], we included this class to provide a set of control stimuli suitable for common experimental designs.

Two conditions have to be satisfied in order to classify a word to one of the 6 classes: (1) the word's distance to the emotion or neutral state must be smaller than a certain threshold; (2) the word must meet the first condition for one category only; in other words, if it falls within an area of intersection of two categories, it remains unclassified. Thus, words more distant from all the classes than the respective thresholds remain unclassified, and so do words that are close (in this sense) to two or more classes.

As for the selection of the thresholds, they can be adjusted to the requirements of a particular experimental design, and may depend on the data to which the classification is being applied. Since the classification of experimental stimuli is pragmatic in nature, there is no need to give absolute significance to particular threshold levels. To facilitate the selection of suitable thresholds, we provide an interactive online tool (http://exp.lobi.nencki.gov.pl/nawl-analysis), which makes it possible to recalculate classes using different values of thresholds or, alternatively, to choose a desired number of words in a class. By using this tool and by examining how the numbers of words in each class change with changing thresholds (S2 Appendix, S3 Appendix), we were able to arrive at a classification that ensures good separation of the classes and reasonable numbers of words in each class. Close examination of the mean ratings for the classes obtained, as well as of the word lists themselves, suggested that good values for the thresholds were: 2.5 for happiness, 5.5 for the remaining basic emotion classes and 2.5 for the neutral class. The threshold for happiness is lower than the thresholds for negative emotions due to the fact that language is positively biased and it is easier to find words that elicit this emotion [35]. The threshold for the neutral class is also lower, which is justified by the fact that the (1,1,1,1,1) point is closer to all the other points (distance of 6) than they are from each other (distance 8.49).

**Fig 2:**
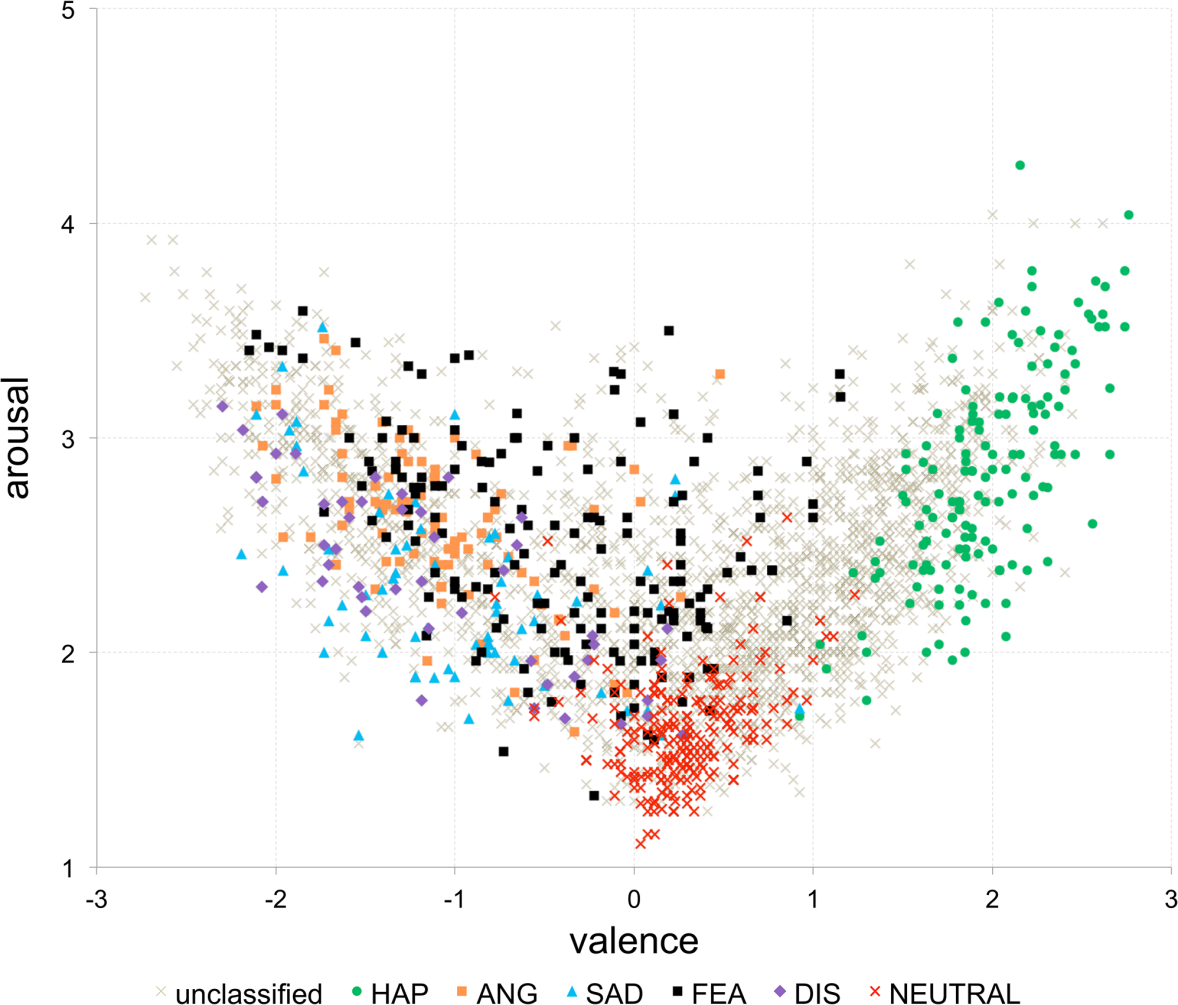
Distribution of the NAWL stimuli assigned to basic emotions in the affective space of valence and arousal. Euclidean distance based classification method was used to classify words (threshold values: 2.5 for happiness, 5.5 for anger, sadness, fear, disgust; 2.5 for the neutral class).

By applying the proposed thresholds we were able to assign 739 out of 2902 words to the following classes: happiness (147), anger (98), sadness (64), fear (163), disgust (48) and neutral (219). With this new data, we compared the “dimensional” framework with the discrete approach by superimposing the sets of NAWL words assigned to basic emotions (identified with our classification method) on the valence-arousal plot shown in Fig 2.

### NAWL and DENN-BAWL comparison

Since the NAWL is an adaptation of the German database, it was natural to ask whether the ratings for each basic emotion obtained in the DENN-BAWL study [7] for German words were correlated with the ratings of the corresponding Polish translations obtained in the NAWL study. Calculations of Pearson’s correlation coefficients were performed only for the stimuli that were included in both databases (n = 1958). All correlations were significant at the .01 level (two-tailed). Strongest correlations were found between the NAWL and the DENN-BAWL ratings of the same basic emotion category (*r* = .81; *p* < 0.01 for happiness; *r* = .78; *p* < 0.01 for anger; *r* = .71; *p* < 0.01 for sadness; *r* = .78; *p* < 0.01 for fear; *r* = .63; *p* < 0.01 for disgust).

Furthermore, we compared the numbers of words in classes generated by the *liberal* and *conservative* criteria [7] for both German and Polish databases (Table 5). Since the remaining method introduced by [16] and based on confidence intervals was never applied to the DENN-BAWL data, we did not compare our database to the DENN-BAWL by the means of this method. Similarly, we were unable to use the new method introduced in the present paper to assess the correspondence between DENN-BAWL and NAWL BE.

**Table 5:** Comparison of the numbers of words classified as basic emotion specific in the NAWL and DENN-BAWL studies.

NAWL (n = 1958)	HAP	ANG	SAD	FEA	DIS	Total
**liberal**	1145	202	109	365	111	**1932**
**conservative**	503	2	7	8	6	**526**
**DENN-BAWL (n = 1958)**						
**liberal**	1104	384	43	261	125	**1917**
**conservative**	430	38	6	6	13	**493**

The analysis revealed that the proportion of words that could be assigned to some emotion category was rather similar for both languages. However, differences in the numbers of words assigned to particular basic emotions were rather striking. German words were more frequently identified as anger-related as compared to Polish words. On the other hand, the proportion of words representing sadness and fear was much greater for Polish words.

## Discussion

Standardized emotional databases of stimuli are an invaluable source of information, as they allow for control and manipulation of experimental conditions. Using validated emotional material, as opposed to stimuli picked by a researcher based on subjective criteria, ensures greater objectivity of inferences. The availability of such databases is also recognized as a more convenient way of conducting research, reducing the time and effort required to prepare an experimental design. Therefore, such instruments are highly regarded and sought after in the research community. The importance of creating new datasets of stimuli is especially important in research on verbal processing, due to differences in connotations attached to words in different languages. In recent years many such word databases were introduced, allowing for the use of such material in English, German, French, Italian, Spanish, Portuguese, Dutch, as well as Polish.

Although both the dimensional and discrete theoretical frameworks for modelling emotions have been widely discussed, most of the standardized datasets provide only information on the dimensional characteristics of emotions associated with words. While some authors [1, 13, 19] have recently called for simultaneous control of dimensional and discrete properties of emotional stimuli, only a few databases have been further developed to allow for the control of category-specific properties [7, 9]. Moreover, recent empirical evidence demonstrated the advantages of such a combined approach, documenting the unique contribution of discrete emotions to an explanation of the affective processing of words [17, 18]. In response to this call, we introduce in this study the basic emotion norms for the Polish language. The NAWL database should prove useful in studies of affect from both perspectives: dimensional and discrete, providing highly controlled experimental verbal material. The general characteristics as well as the relationships between the variables included in the NAWL word database are congruent with typical patterns found in previous studies [7, 9]. In particular, like the authors of the German database [7], we found basic emotions to be more strongly correlated with valence than with arousal.

Moreover, a comparison of the NAWL stimuli and their German counterparts from the DENN-BAWL [7] revealed that ratings obtained from the two studies are largely similar. However, we make no attempt to explain the relation between the NAWL BE ratings and the English study [9]. Since the NAWL dataset was developed as a cultural adaptation of the BAWL-R [23], its content fully corresponds to the material included in the German database. In fact, the ANEW [9] database was already adapted to German and the norms for several affective variables (valence, arousal, dominance, imageability, potency) were published as the ANGST database [32]. However, the authors found that only 400 words from the ANGST were included in the BAWL-R. Hence, there is only a small overlap between the ANEW and the BAWL. We decided that it would be misleading to compare the present dataset and 400 words from the ANEW. However, based on the comparison between the NAWL BE and DENN-BAWL we expect that Polish words may be successfully used to induce different types of basic emotions.

To make the database more useful as a research tool, several classification methods were applied to the NAWL ratings, in order to identify words that specifically elicited a particular category of emotion [7, 16]. We supplemented these methods with one of our own. The classification we propose has several advantages over the existing methods. First of all, by converting raw ratings to Euclidean distances we were able to distinguish not only classes representing basic emotions, but also words most closely related to the neutral state. Although previous classification methods neglected this class, we found it useful as a source of control experimental stimuli. Moreover, average Euclidean distances–as opposed to mean ratings–better capture the association of words with ‘pure’ basic emotions and take into account correlations between the ratings obtained for different emotions. Secondly, we found the existing classification methods not entirely satisfactory, being either too broad or too narrow. Compared to the criteria previously introduced [7, 16], the criterion based on Euclidean distances allows the researcher to create longer lists of words likely to be related to a single basic emotion—including anger, sadness, fear and disgust, which are relatively difficult to elicit with verbal stimuli. Another advantage of our method is its flexibility, since its parameters can be adjusted to the stimuli as well as to the purposes of a particular research design. Nevertheless, we identified thresholds optimal for the use of the NAWL ratings and classified words according to them. In doing so, we wanted to maximise the number of words assigned to classes, without compromising the quality of the classes created. One should bear in mind that there is always a trade-off between these two aims. The complete ratings together with the respective classification labels are available for research and non-commercial use and can be found in the supplementary online materials.

### Limitations and future directions

Since subjects were instructed to report their actual emotional state, most words were assigned rather low emotion values. As previous studies indicated [36] this is quite distinctive of verbal material (as contrasted to non-verbal material). This problem was especially apparent in the case of anger, sadness, fear and disgust. Thus, any differentiation of words related to specific basic emotions should necessarily result in disproportions (either in terms of number or quality) between the class of happiness-eliciting words and the remaining classes. Nevertheless, with the classification method introduced here, researchers will be able to make proper decisions regarding the classification outcome that best suits their needs. In order to make this decision process more convenient, we provide a web-based interface, allowing the user to generate classes of stimuli that meet the desired criteria.

Ratings presented here are an initial approximation of the emotional impact of the NAWL stimuli, albeit an indispensable one, due to a great amount of verbal material that has to be sifted through. More reliable measures of the power of words to elicit emotions, involving objective physiological and neuroimaging methods, can now be used with informed guidance provided by data described here. In particular, usefulness of the original BAWL-R [23] and DENN-BAWL [7] datasets has been already widely demonstrated (see [19] for a review). On these grounds, we expect that by making analogous Polish verbal material available to the scientific community, we will encourage similar attempts to further validate the NAWL database empirically and to pursue cross-cultural comparisons. In any case, our database provides a fairly comprehensive catalogue of the affective features of verbal stimuli for the Polish language.

### Description of the database

The supplementary material can be found in S1 Dataset. The material is organized as follows:

**No.**—Number identifying each of the 2902 words and corresponding to the original number in the BAWL-R [23]
**BAWL_word**—Original German words from the BAWL-R [23]
**NAWL_word**—Polish words in alphabetical order
**BE_N_all**—Total number of ratings of basic emotions
**hap_M_all**—Mean (M) of happiness ratings, ranging from 1 to 7, where: 1 = low intensity, 7 = strong intensity
**hap_SD_all**—Standard deviation (SD) of happiness ratings
**ang_M_all**—Mean (M) of anger ratings, ranging from 1 to 7, where: 1 = low intensity, 7 = strong intensity
**ang_SD_all**—Standard deviation (SD) of anger ratings
**sad_M_all**—Mean (M) of sadness ratings, ranging from 1 to 7, where: 1 = low intensity, 7 = strong intensity
**sad_SD_all**—Standard deviation (SD) of sadness ratings
**fea_M_all**—Mean (M) of fear ratings, ranging from 1 to 7, where: 1 = low intensity, 7 = strong intensity
**fea_SD_all**—Standard deviation (SD) of fear ratings
**dis_M_all**—Mean (M) of disgust ratings, ranging from 1 to 7, where: 1 = low intensity, 7 = strong intensity
**dis_SD_all**—Standard deviation (SD) of disgust ratings
**BE_N_women**—Number of ratings of basic emotions given by women
**hap_M_women**—Mean (M) of happiness ratings given by females, ranging from 1 to 7, where: 1 = low intensity, 7 = strong intensity
**hap_SD_women**—Standard deviation (SD) of happiness ratings given by females
**ang_M_women**—Mean (M) of anger ratings given by females, ranging from 1 to 7, where: 1 = low intensity, 7 = strong intensity
**ang_SD_women**—Standard deviation (SD) of anger ratings given by females
**sad_M_women**—Mean (M) of sadness ratings given by females, ranging from 1 to 7, where: 1 = low intensity, 7 = strong intensity
**sad_SD_women**—Standard deviation (SD) of sadness ratings given by females
**fea_M_women**—Mean (M) of fear ratings given by females, ranging from 1 to 7, where: 1 = low intensity, 7 = strong intensity
**fea_SD_women**—Standard deviation (SD) of fear ratings given by females
**dis_M_women**—Mean (M) of disgust ratings given by females, ranging from 1 to 7, where: 1 = low intensity, 7 = strong intensity
**dis_SD_women**—Standard deviation (SD) of disgust ratings given by females
**BE_N_men**—Number of ratings of basic emotions given by men
**hap_M_men**—Mean (M) of happiness ratings given by males, ranging from 1 to 7, where: 1 = low intensity, 7 = strong intensity
**hap_SD_men**—Standard deviation (SD) of happiness ratings given by males
**ang_M_men**—Mean (M) of anger ratings given by males, ranging from 1 to 7, where: 1 = low intensity, 7 = strong intensity
**ang_SD_men—**Standard deviation (SD) of anger ratings given by males
**sad_M_men**—Mean (M) of sadness ratings given by males, ranging from 1 to 7, where: 1 = low intensity, 7 = strong intensity
**sad_SD_men**—Standard deviation (SD) of sadness ratings given by males
**fea_M_men**—Mean (M) of fear ratings given by males, ranging from 1 to 7, where: 1 = low intensity, 7 = strong intensity
**fea_SD_men**—Standard deviation (SD) of fear ratings given by males
**dis_M_men**—Mean (M) of disgust ratings given by males, ranging from 1 to 7, where: 1 = low intensity, 7 = strong intensity
**dis_SD_men**—Standard deviation (SD) of disgust ratings given by males
**DENN_BAWL**—Inclusion of German words from BAWL-R in DENN_BAWL dataset of basic emotion norms
**Briesemeister_liberal**—Basic emotion classification according to DENN_BAWL liberal criterion [7]
**Briesemeister_conservative** - Basic emotion classification according to DENN_BAWL conservative criterion [7]
**Mikels**—Basic emotion classification based on the confidence intervals (CI) criterion [16]
**Mikels_blended**—Basic emotion classification based on the confidence intervals (CI) criterion [16]—blended emotions specified
**ED_class**—Basic emotion classification based on the Euclidean distance (ED) criterion (threshold of 2.5 for happiness and the neutral state, and 5.5 for the remaining emotions)

## Supporting Information

S1 Appendix Instruction for basic emotions rating procedure in the NAWL study: Polish original and English translation. (DOCX)

S2 Appendix Number of words in the NAWL database assigned to particular categories depending on threshold values (equal for all categories). (PNG)

S3 Appendix Optimal threshold values for the happiness and neutral classes, given a fixed threshold for the remaining classes (5.5). (PNG)

S1 Dataset Affective ratings and basic emotions classification labels for 2902 Polish words. The database includes happiness, anger, sadness, fear and disgust ratings separately for the whole sample, as well as for females and males separately. The detailed description of the contents of the database can be found in the Description of the database section.
(XLSX)
