# Supplementary material for: Basic Emotions in the Nencki Affective Word List (NAWL BE): New Method of Classifying Emotional Stimuli
Source: PLoS One. 2015 Jul 6;10(7):e0132305. doi: 10.1371/journal.pone.0132305 (PMC4492597; doi:10.1371/journal.pone.0132305)
Supplement: S1 Appendix — (DOCX) [file pone.0132305.s001.docx]

*Instrukcja*

*Dziękujemy za Twoją zgodę na udział w badaniu.*

*Badanie dotyczy reakcji emocjonalnych na słowa. Przez około 60 minut będziesz czytać słowa wyświetlane na ekranie komputera. Twoim zadaniem będzie ocenić każde z 291 słów na pięciu skalach, reprezentujących podstawowe rodzaje emocji: szczęście, złość, smutek, strach, oraz wstręt.*

*Będziesz musiał/a określić, w jakim stopniu dane słowo wzbudza (wywołuje) w Tobie dany rodzaj emocji, wskazując na każdej ze skal ocenę od 1 do 7, gdzie:*

*1 = to słowo wcale, w niewielkim stopniu budzi we mnie daną emocję*

*7 = to słowo bardzo, w znacznym stopniu budzi we mnie daną emocję*

*Po ocenieniu połowy słów czeka Cię krótka przerwa.*

*Nie ma dobrych, ani złych odpowiedzi; odpowiadaj zgodnie z pierwszym skojarzeniem i staraj się wykorzystywać cały zakres skali ocen. W dowolnym momencie możesz wrócić do instrukcji (poprzez naciśnięcie odnośnika w prawym górnym rogu), a potem kontynuować badanie.*

*Czytanie niektórych słów może się wydać nieprzyjemne. Gdybyś poczuł/a, że nie chcesz kontynuować badania, możesz je przerwać w dowolnym momencie. W przypadku pytań, zwróć się do eksperymentatora.*

*Instructions*

*Thank you for your participation in the experiment.*

*This is a study of emotional reactions to words. During the session, which will last for about 60 minutes, you will be presented with words on the computer screen. Your task is to rate each of the 291 words on five scales, corresponding to the basic types of emotion: happiness, anger, sadness, fear and disgust.*

*Please, assess the degree to which the displayed word evokes in you a given type of emotion by rating it on a scale from 1 to 7, where:*

*1 = this word does not evoke this emotion in me at all / only slightly evokes this emotion in me*

*7 = this word strongly evokes this emotion in me / evokes this emotion in me to a significant extent*

*There will be a short break in the middle of the session.*

*There is no right or wrong answer; choose the answer that comes to mind first and try to use the whole range of values. You can return to the instruction at any time by clicking a link in the upper right-hand corner, and then resume work.*

*Reading some of the words may be unpleasant. You may stop at any point, should you feel you would rather not continue the experiment. If you have any questions, please ask the assistant.*
