# Supplementary figures and images for: Basic Emotions in the Nencki Affective Word List (NAWL BE): New Method of Classifying Emotional Stimuli
Source: PLoS One. 2015 Jul 6;10(7):e0132305. doi: 10.1371/journal.pone.0132305 (PMC4492597; doi:10.1371/journal.pone.0132305)

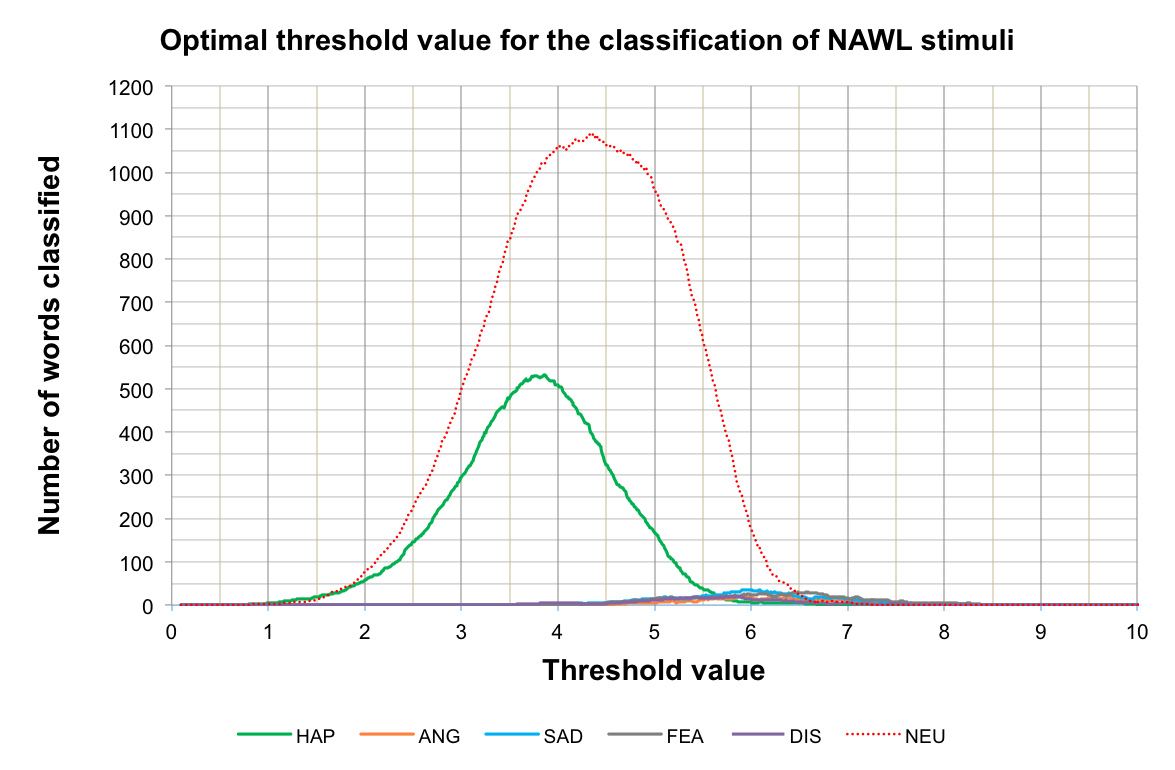

Supplement: S2 Appendix — (PNG) [file pone.0132305.s002.png]

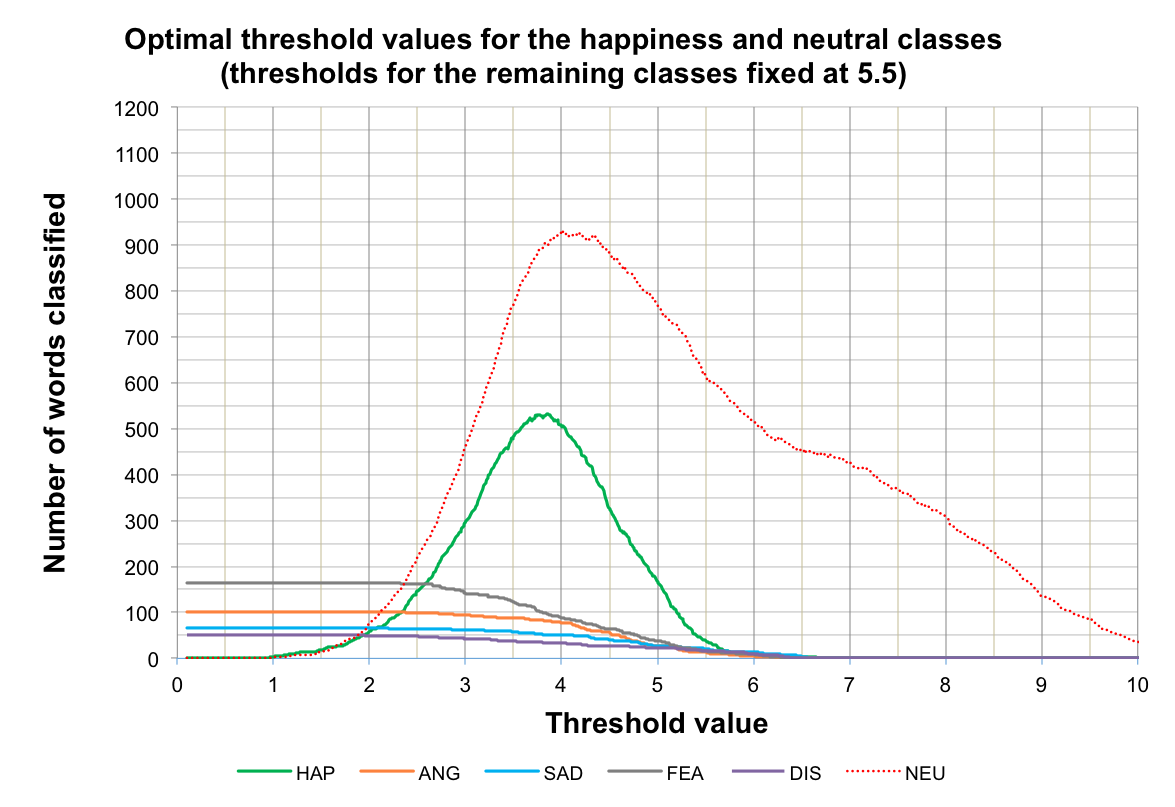

Supplement: S3 Appendix — (PNG) [file pone.0132305.s003.png]
